# Association between gestational weight gain and adverse neonatal outcomes in women conceiving with assisted reproductive technology: Evidence from the NVSS 2019–2021

**DOI:** 10.1371/journal.pone.0292665

**Published:** 2023-10-26

**Authors:** Feifei Jiang, Yanan Li, Lipeng Sun

**Affiliations:** 1 Department of Neonatology, Xiamen Humanity Maternity Hospital, Xiamen, Fujian, China; 2 Department of Neonatology, Bozhou People’s Hospital, Bozhou, Anhui, China; University of Catania: Universita degli Studi di Catania, ITALY

## Abstract

**Objective:**

To evaluate the association between gestational weight gain (GWG) and adverse neonatal outcomes in women who conceived using assisted reproductive technology (ART).

**Methods:**

The National Vital Statistics System (NVSS) 2019–2021 provided data for this retrospective cohort study. Adverse neonatal outcomes included premature birth, small for gestational age (SGA), large for gestational age (LGA), macrosomia, low birth weight (LBW), and other abnormal conditions. Any adverse outcome was defined as at least one of the above six outcomes. Multivariate logistic regression analysis was employed to evaluate the associations between GWG and different outcomes, after adjusting for confounding factors. These associations were further assessed in subgroups of maternal age at delivery, paternal age at delivery, preconception body mass index (BMI), gestational age, maternal race, parity, gestational diabetes, and gestational hypertension.

**Results:**

Totally 108201 women were included, with 22282 in the insufficient GWG group, 38034 in the sufficient GWG group, and 47885 in the excessive GWG group. Women with insufficient GWG [odds ratios (OR) = 1.11, 95%CI: 1.07–1.16, *P*<0.001] and excessive GWG (OR = 1.14, 95%CI: 1.10–1.18, *P*<0.001) had significantly greater risks of any adverse outcome than those with sufficient GWG. In contrast to sufficient GWG, insufficient GWG was associated with significantly elevated risks of premature birth (OR = 1.42, 95%CI: 1.35–1.48, *P*<0.001), SGA (OR = 1.45, 95%CI: 1.37–1.53, *P*<0.001), LBW (OR = 1.47, 95%CI: 1.37–1.58, *P*<0.001), and other abnormal conditions (OR = 1.32, 95%CI: 1.27–1.39, *P*<0.001), and excessive GWG was associated with significantly lower risks of premature birth (OR = 0.86, 95%CI: 0.83–0.90, *P*<0.001), SGA (OR = 0.79, 95%CI: 0.75–0.83, *P*<0.001), LBW (OR = 0.85, 95%CI: 0.79–0.91, *P*<0.001), and other abnormal conditions (OR = 0.92, 95%CI: 0.88–0.96, *P*<0.001). Infants born to women with insufficient GWG had significantly decreased risks of LGA (OR = 0.71, 95%CI: 0.66–0.75, *P*<0.001) and macrosomia (OR = 0.68, 95%CI: 0.63–0.74, *P*<0.001), and infants born to women with excessive GWG had significantly increased risks of LGA (OR = 1.50, 95%CI: 1.44–1.56, *P*<0.001) and macrosomia (OR = 1.60, 95%CI: 1.51–1.69, *P*<0.001).

**Conclusion:**

Insufficient GWG and excessive GWG were associated with increased risks of any adverse outcome than sufficient GWG in women who conceived with ART, indicating the applicability of recommended GWG by the Institute of Medicine (IOM) in this population.

## Introduction

With the increasing prevalence of infertility and the postponement of childbearing time worldwide, the application of assisted reproductive technology (ART) is gradually increasing [1, 2], which has become an integral part of modern medicine and now plays a critical role in realizing family planning [3]. At least 5 million babies have been born resulting from ART. In some countries, the proportion of babies born following ART now exceeds 5% [3]. In the past, reproductive experts focused on how to improve the clinical pregnancy rate and cumulative live birth rate. However, some non-physiological interventions during ART, especially the use of hormone drugs with supraphysiological doses, may affect the overall environment of pregnancy, interfere with gametogenesis or embryonic development, and adversely influence the outcomes of mothers and newborns. Evidence has shown that compared with spontaneous pregnancies, ART-induced pregnancies have an increased risk of adverse neonatal outcomes, such as preterm birth (10.9% vs 6.4%), low birth weight (LBW) (8.7% vs 5.8%), small for gestational age (SGA) (7.1% vs 5.7%), and perinatal mortality (1.1% vs 0.6%) for singleton conceptions following in vitro fertilization/intracytoplasmic sperm injection (IVF/ICSI) [4–7]. Hence, more attention should be paid to pregnancies conceived via ART.

Gestational weight gain (GWG) is a potentially modifiable factor in adverse pregnancy outcomes [8]. GWG is related to maternal fat accumulation, fetal, placental, and uterine growth, etc., and is essential to ensure fetal health, but excessive or insufficient increase can lead to an elevated risk of adverse outcomes. Preconception body mass index (BMI) is associated with GWG and the risk of adverse pregnancy outcomes, and thus, the determination of the best range of GWG should consider preconception BMI [9, 10]. The 2009 Institute of Medicine (IOM) guidelines determined the optimal range of GWG for singleton pregnancy under different preconception BMI conditions [11, 12]. At present, the association of preconception BMI and GWG with the pregnancy outcomes of women conceiving with ART and the applicability of the GWG recommended by the IOM in this population have not been clarified.

This study intended to evaluate the association between GWG and adverse neonatal outcomes in women who conceived using ART, considering preconception BMI and using the data from the National Vital Statistics System (NVSS) database, in order to assess the applicability of the GWG recommended by the IOM in this population. The association was further assessed in subgroups of maternal age at delivery, paternal age at delivery, preconception BMI, gestational age, maternal race, parity, gestational diabetes, and gestational hypertension.

## Methods

### Study design and population

The NVSS 2019–2021 provided data for this retrospective cohort study. The NVSS database collects nationwide data on births, deaths, marriages, and other events in 50 states, New York City, District of Columbia, and 5 territories (Puerto Rico, Virgin Islands, Guam, American Samoa, and Northern Mariana Islands) of the United States [13]. Since these data are publicly available and de-identified, this study was exempt from institutional review board approval. The study involved women (1) aged ≥18 years, (2) with singleton pregnancy resulting from ART, (3) whose babies were born at 24–42 weeks of gestational age, (4) with complete information on pre-pregnancy maternal weight, maternal height, and maternal weight at the time of delivery. Women (1) with multifetal pregnancies or stillbirths, (2) with missing information on neonatal outcomes, (3) with missing information on key covariates were excluded. The follow-up was ended at the birth of newborns.

### Preconception BMI and GWG

Preconception BMI was classified according to World Health Organization (WHO) standards: BMI < 18.5 kg/m^2^ was considered as underweight, BMI of 18.5–24.9 kg/m^2^ as normal weight, BMI of 25.0–29.9 kg/m^2^ as overweight, and BMI ≥ 30.0 kg/m^2^ as obese, of which 30.0–34.9 kg/m^2^ was regarded as obesity class I, 35.0–39.9 kg/m^2^ as obesity class II, and ≥ 40 kg/m^2^ as obesity class III. For GWG classification, when preconception BMI < 18.5 kg/m^2^ (underweight), 12.5–18.0 kg was regarded as sufficient GWG; when preconception BMI was 18.5–24.9 kg/m^2^ (normal weight), 11.5–16.0 kg was regarded as sufficient GWG; when preconception BMI was 25.0–29.9 kg/m^2^ (overweight), 7.0–11.5 kg was regarded as sufficient GWG; when preconception BMI ≥ 30.0 kg/m^2^ (obese), 5.0–9.0 kg was regarded as sufficient GWG. The weight gain below sufficient GWG was deemed as insufficient weight. The weight gain above sufficient GWG was considered as excessive weight [11, 12].

### Adverse neonatal outcomes

Adverse neonatal outcomes included premature birth, SGA, large for gestational age (LGA), macrosomia, LBW, and other abnormal conditions [assisted ventilation required immediately following delivery, assisted ventilation required for more than six hours, Neonatal Intensive Care Unit (NICU) admission, newborn given surfactant replacement therapy, antibiotics received by the newborn for suspected neonatal sepsis, seizure or serious neurologic dysfunction]. Premature birth was defined as birth at gestational age < 37 weeks. SGA was defined as a birth weight below the 10th percentile. LGA was defined as a birth weight above the 90th percentile. Macrosomia was defined as a birth weight > 4000 g. LBW was defined as a birth weight < 2500 g. Any adverse outcome was defined as at least one of the above six outcomes.

### Data collection

The following clinical data were collected: gestational age (weeks), maternal age at delivery (years), maternal race (White, Black, Asian, Other), maternal education level (high school or above, less than high school, other\unknown), paternal age at delivery, paternal education level (high school or above, less than high school, other\unknown), paternal race (White, Black, Asian, Other), marital status (married, unmarried), parity (multipara, nullipara, unknown), smoking before pregnancy, smoking during pregnancy, start time of prenatal care (months), prenatal care visit (times), pre-gestational diabetes, gestational diabetes, pre-gestational hypertension, gestational hypertension, hypertension eclampsia, fever, previous premature birth, previous cesarean delivery.

### Statistical analysis

Continuous data with normal distribution were reported as mean ± standard deviation (Mean ± SD), and the t test was used for comparisons among groups. Continuous data with skewed distribution described with median and quartile [M (Q_1_, Q_3_)], and the rank sum test was applied for intergroup comparisons. Categorical data were shown as the number of cases and the composition ratio [n (%)], and intergroup comparisons were subject to the Chi-square test. Missing values were deleted, and sensitivity analysis was performed for data before and after deletion of missing values (S1 Table). Univariate logistic regression analysis was used for covariate screening (S2 Table). The associations between GWG and different neonatal outcomes were evaluated via univariate and multivariate logistic regression analysis. Model 1 did not adjust for covariates. Model 2 adjusted for maternal age at delivery, maternal race, and maternal education level. Model 3 adjusted for maternal age at delivery, maternal race, maternal education level, paternal age at delivery, paternal race, and paternal education level. Model 4 adjusted for maternal age at delivery, maternal race, maternal education level, paternal age at delivery, paternal race, paternal education level, parity, smoking before pregnancy, smoking during pregnancy, prenatal care visit, pre-gestational diabetes, gestational diabetes, pre-gestational hypertension, gestational hypertension, hypertension eclampsia, fever, previous premature birth, previous cesarean delivery, and gestational age (gestational age was not adjusted for when premature birth was the outcome). The associations between GWG and different outcomes were further assessed in subgroups of maternal age at delivery, paternal age at delivery, preconception BMI, gestational age, maternal race, parity, gestational diabetes, and gestational hypertension. Odds ratios (ORs) and 95% confidence intervals (CIs) were calculated. SAS 9.4 (SAS Institute Inc., Cary, NC, USA) was utilized for data extraction and cleaning. Statistical analysis was conducted with R 4.2.0 (R Foundation for Statistical Computing, Vienna, Austria). *P*<0.05 denoted a statistically significant difference.

## Results

### Characteristics of the study population

**Fig 1:**
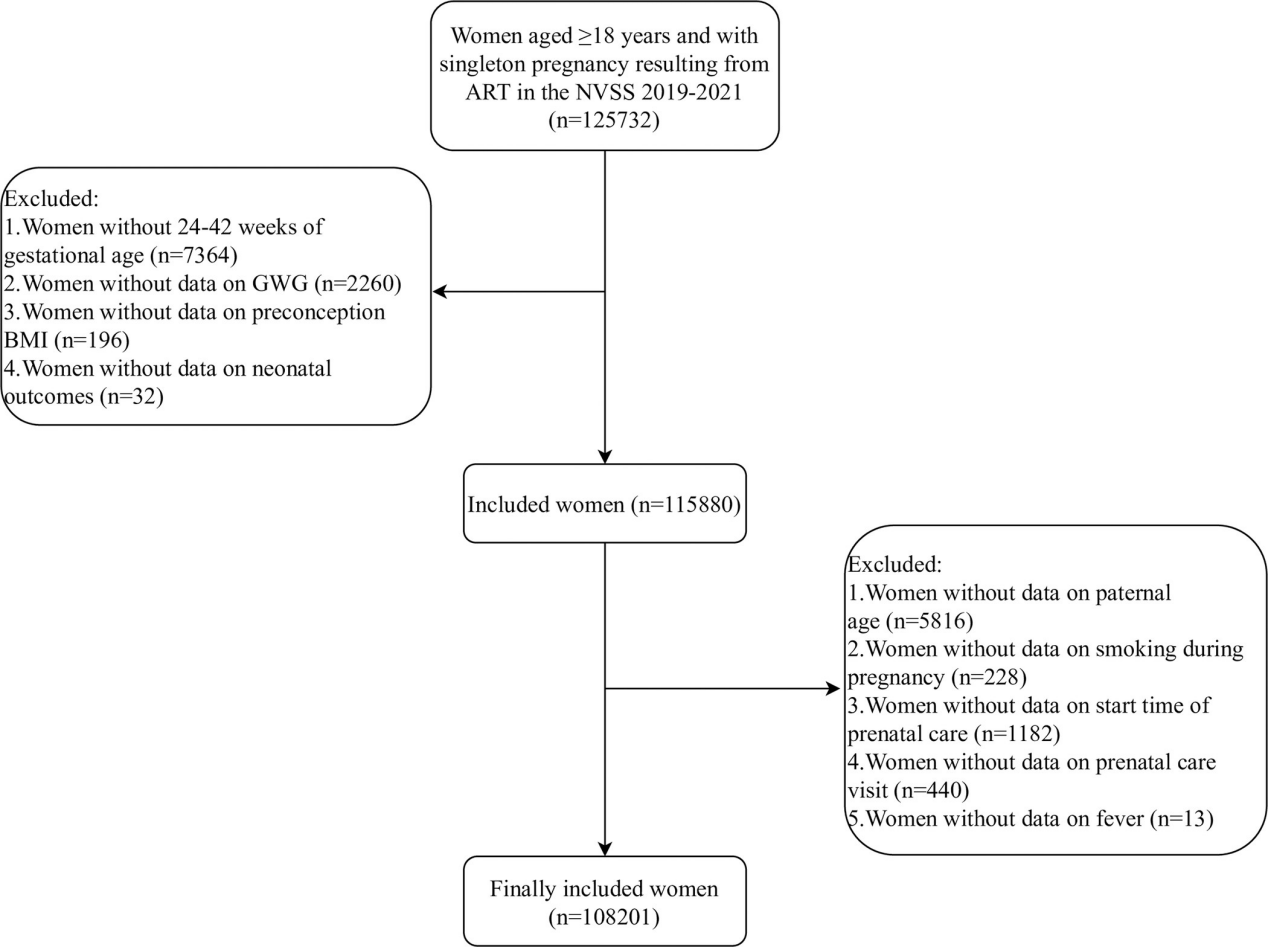
Flow chart of study population selection. NVSS, the National Vital Statistics System; ART, assisted reproductive technology; GWG, gestational weight gain; BMI, body mass index.

In total, 125732 women aged ≥18 years and with singleton pregnancy resulting from ART were included from the NVSS 2019–2021. After excluding women without 24–42 weeks of gestational age (n = 7364), and with missing information on GWG (n = 2260), preconception BMI (n = 196), neonatal outcomes (n = 32), paternal age (n = 5816), smoking during pregnancy (n = 228), start time of prenatal care (n = 1182), prenatal care visit (n = 440), and fever (n = 13), 108201 women were eligible for this study. The flow chart of study population selection is illustrated in Fig 1. Most of participants (48.57%) had normal preconception BMI. The average gestational age and maternal age at delivery were 38.21 weeks and 35.14 years. The majority of women were Whites (77.32%). Table 1 demonstrates the characteristics of the study population. Significant differences were found in preconception BMI, GWG, gestational age, maternal age at delivery, maternal race, maternal education level, paternal age at delivery, paternal race, paternal education level, parity, smoking before pregnancy, smoking during pregnancy, start time of prenatal care, prenatal care visit, pre-gestational diabetes, gestational diabetes, pre-gestational hypertension, gestational hypertension, hypertension eclampsia, previous premature birth, previous cesarean delivery among insufficient (n = 22282), sufficient (n = 38034), and excessive (n = 47885) GWG groups (all *P*<0.05).

**Table 1:** Characteristics of the study population.

Variables	Total (n = 108201)	GWG			*P*
		Insufficient (n = 22282)	Sufficient (n = 38034)	Excessive (n = 47885)	
Preconception BMI, n (%)					<0.001
Underweight	2315 (2.14)	995 (4.47)	1003 (2.64)	317 (0.66)	
Normal	52549 (48.57)	13470 (60.45)	22694 (59.67)	16385 (34.22)	
Overweight	28719 (26.54)	3192 (14.33)	8185 (21.52)	17342 (36.22)	
Obesity I	24618 (22.75)	4625 (20.76)	6152 (16.18)	13841 (28.90)	
GWG, pounds, M (Q_1_, Q_3_)	29.00 (21.00, 37.00)	16.00 (9.00, 21.00)	27.00 (20.00, 31.00)	38.00 (31.00, 45.00)	<0.001
Gestational age, weeks, Mean ± SD	38.21 ± 2.17	37.85 ± 2.58	38.27 ± 2.11	38.33 ± 1.98	<0.001
Maternal age at delivery, years, Mean ± SD	35.14 ± 4.90	35.53 ± 5.08	35.24 ± 4.83	34.89 ± 4.87	<0.001
Maternal race, n (%)					<0.001
White	83664 (77.32)	15773 (70.79)	28932 (76.07)	38959 (81.36)	
Black	5956 (5.50)	1322 (5.93)	1808 (4.75)	2826 (5.90)	
Asian	14379 (13.29)	4226 (18.97)	5794 (15.23)	4359 (9.10)	
Other	4202 (3.88)	961 (4.31)	1500 (3.94)	1741 (3.64)	
Maternal education level, n (%)					<0.001
High school or above	104131 (96.24)	21305 (95.62)	36666 (96.40)	46160 (96.40)	
Less than high school	948 (0.88)	234 (1.05)	312 (0.82)	402 (0.84)	
Other\unknown	3122 (2.89)	743 (3.33)	1056 (2.78)	1323 (2.76)	
Paternal age at delivery, years, Mean ± SD	38.13 ± 6.39	38.50 ± 6.58	38.20 ± 6.34	37.91 ± 6.32	<0.001
Paternal education level, n (%)					0.004
High school or above	103130 (95.31)	21143 (94.89)	36353 (95.58)	45634 (95.30)	
Less than high school	1484 (1.37)	332 (1.49)	497 (1.31)	655 (1.37)	
Other\unknown	3587 (3.32)	807 (3.62)	1184 (3.11)	1596 (3.33)	
Paternal race, n (%)					<0.001
White	81743 (75.55)	15844 (71.11)	28552 (75.07)	37347 (77.99)	
Black	6028 (5.57)	1249 (5.61)	1823 (4.79)	2956 (6.17)	
Asian	13195 (12.19)	3671 (16.48)	5218 (13.72)	4306 (8.99)	
Other	7235 (6.69)	1518 (6.81)	2441 (6.42)	3276 (6.84)	
Marital status, n (%)					0.082
Married	87455 (95.39)	17669 (95.33)	30278 (95.60)	39508 (95.25)	
Unmarried	4230 (4.61)	865 (4.67)	1395 (4.40)	1970 (4.75)	
Parity, n (%)					<0.001
Multipara	32461 (30.00)	6976 (31.31)	11705 (30.78)	13780 (28.78)	
Nullipara	62949 (58.18)	12690 (56.95)	21993 (57.82)	28266 (59.03)	
Unknown	12791 (11.82)	2616 (11.74)	4336 (11.40)	5839 (12.19)	
Smoking before pregnancy, n (%)					<0.001
No	107440 (99.30)	22152 (99.42)	37850 (99.52)	47438 (99.07)	
Yes	761 (0.70)	130 (0.58)	184 (0.48)	447 (0.93)	
Smoking during pregnancy, n (%)					0.008
No	107881 (99.70)	22220 (99.72)	37944 (99.76)	47717 (99.65)	
Yes	320 (0.30)	62 (0.28)	90 (0.24)	168 (0.35)	
Start time of prenatal care, months, M (Q_1_, Q_3_)	3.00 (2.00, 3.00)	3.00 (2.00, 3.00)	3.00 (2.00, 3.00)	3.00 (2.00, 3.00)	0.003
Prenatal care visit, times, M (Q_1_, Q_3_)	12.00 (10.00, 14.00)	12.00 (10.00, 14.00)	12.00 (10.00, 14.00)	12.00 (10.00, 14.00)	<0.001
Pre-gestational diabetes, n (%)					<0.001
No	106907 (98.80)	21975 (98.62)	37645 (98.98)	47287 (98.75)	
Yes	1294 (1.20)	307 (1.38)	389 (1.02)	598 (1.25)	
Gestational diabetes, n (%)					<0.001
No	95103 (87.89)	18377 (82.47)	33624 (88.41)	43102 (90.01)	
Yes	13098 (12.11)	3905 (17.53)	4410 (11.59)	4783 (9.99)	
Pre-gestational hypertension, n (%)					<0.001
No	104287 (96.38)	21374 (95.92)	36870 (96.94)	46043 (96.15)	
Yes	3914 (3.62)	908 (4.08)	1164 (3.06)	1842 (3.85)	
Gestational hypertension, n (%)					<0.001
No	94595 (87.43)	20168 (90.51)	34037 (89.49)	40390 (84.35)	
Yes	13606 (12.57)	2114 (9.49)	3997 (10.51)	7495 (15.65)	
Hypertension eclampsia, n (%)					0.004
No	107854 (99.68)	22226 (99.75)	37927 (99.72)	47701 (99.62)	
Yes	347 (0.32)	56 (0.25)	107 (0.28)	184 (0.38)	
Fever, n (%)					0.108
No	104867 (96.92)	21636 (97.10)	36815 (96.79)	46416 (96.93)	
Yes	3334 (3.08)	646 (2.90)	1219 (3.21)	1469 (3.07)	
Previous premature birth, n (%)					<0.001
No	104592 (96.66)	21451 (96.27)	36829 (96.83)	46312 (96.72)	
Yes	3609 (3.34)	831 (3.73)	1205 (3.17)	1573 (3.28)	
Previous cesarean delivery, n (%)					<0.001
No	91309 (84.39)	18982 (85.19)	32220 (84.71)	40107 (83.76)	
Yes	16892 (15.61)	3300 (14.81)	5814 (15.29)	7778 (16.24)	
Any adverse outcome, n (%)					<0.001
No	64119 (59.26)	12752 (57.23)	23634 (62.14)	27733 (57.92)	
Yes	44082 (40.74)	9530 (42.77)	14400 (37.86)	20152 (42.08)	
Premature birth, n (%)					<0.001
No	92332 (85.33)	18053 (81.02)	32744 (86.09)	41535 (86.74)	
Yes	15869 (14.67)	4229 (18.98)	5290 (13.91)	6350 (13.26)	
LGA, n (%)					<0.001
No	92810 (85.78)	20387 (91.50)	33731 (88.69)	38692 (80.80)	
Yes	15391 (14.22)	1895 (8.50)	4303 (11.31)	9193 (19.20)	
SGA, n (%)					<0.001
No	98277 (90.83)	19154 (85.96)	34377 (90.38)	44746 (93.44)	
Yes	9924 (9.17)	3128 (14.04)	3657 (9.62)	3139 (6.56)	
Macrosomia, n (%)					<0.001
No	99188 (91.67)	21462 (96.32)	35699 (93.86)	42027 (87.77)	
Yes	9013 (8.33)	820 (3.68)	2335 (6.14)	5858 (12.23)	
LBW, n (%)					<0.001
No	99645 (92.09)	19355 (86.86)	35191 (92.53)	45099 (94.18)	
Yes	8556 (7.91)	2927 (13.14)	2843 (7.47)	2786 (5.82)	
Other abnormal conditions, n (%)					<0.001
No	90680 (83.81)	18026 (80.90)	32247 (84.78)	40407 (84.38)	
Yes	17521 (16.19)	4256 (19.10)	5787 (15.22)	7478 (15.62)	

BMI, body mass index; GWG, gestational weight gain; LGA, large for gestational age; SGA, small for gestational age; LBW, low birth weight; SD, standard deviation; M, Median; Q_1_, 1st quartile; Q_3_, 3st quartile.

### Relationship between GWG and adverse neonatal outcomes

**Fig 2:**
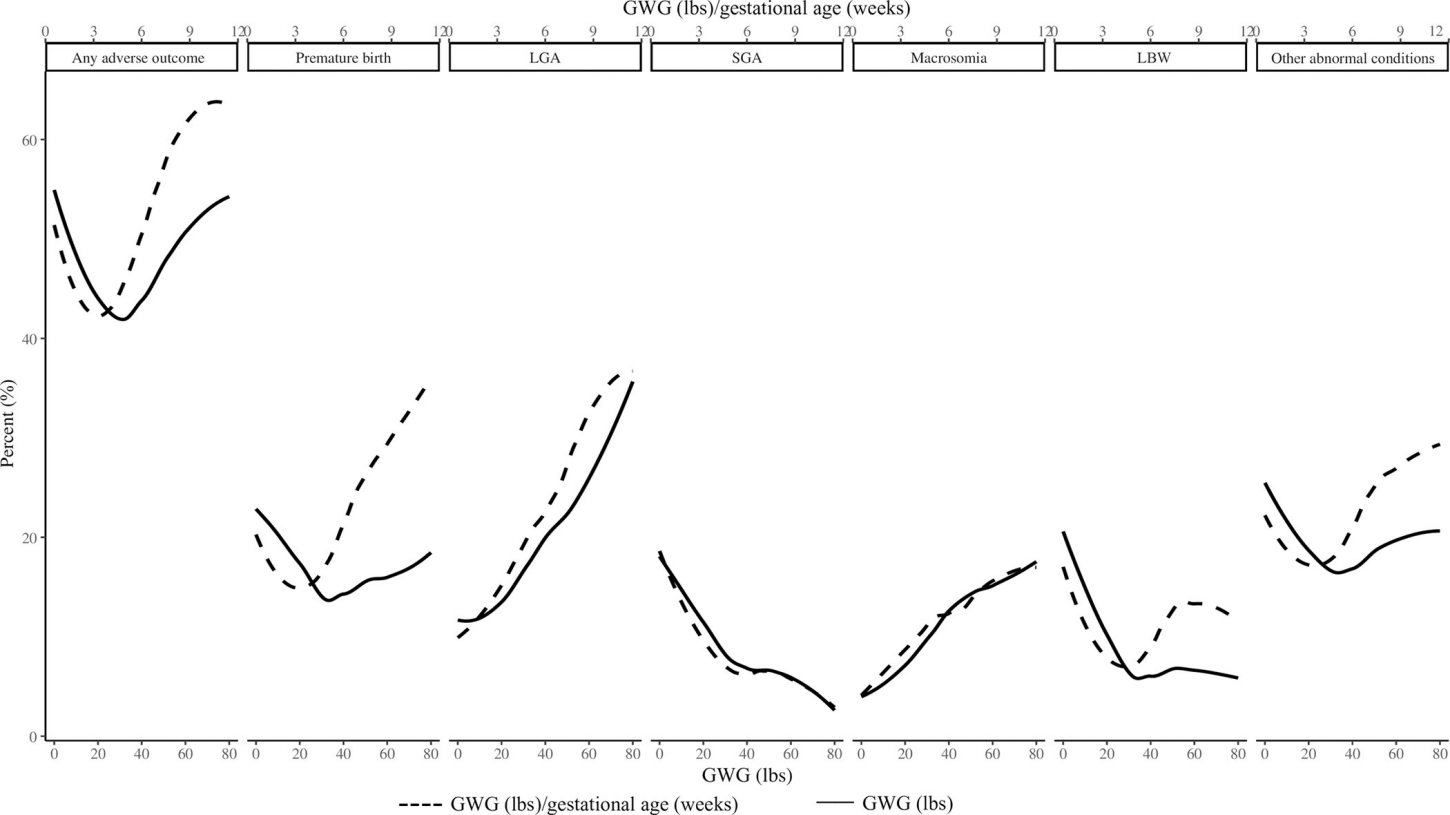
Relationship between GWG and adverse neonatal outcomes. GWG, gestational weight gain; LGA, large for gestational age; SGA, small for gestational age; LBW, low birth weight.

**Fig 3:**
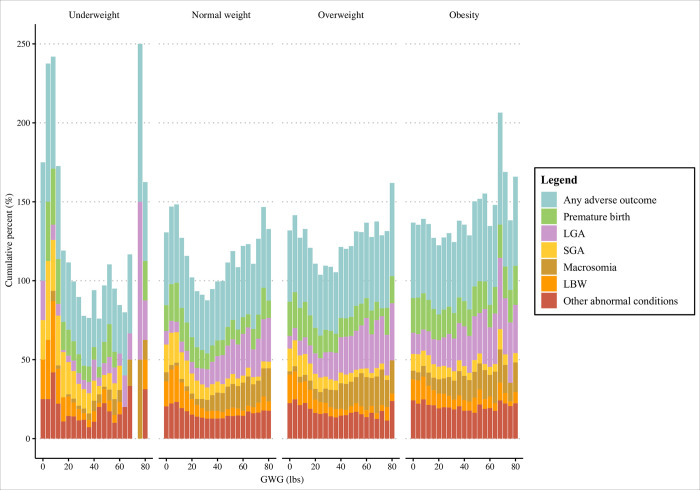
Relationship between GWG and adverse neonatal outcomes in different preconception BMI groups. BMI, body mass index; GWG, gestational weight gain; LGA, large for gestational age; SGA, small for gestational age; LBW, low birth weight.

**Fig 4:**
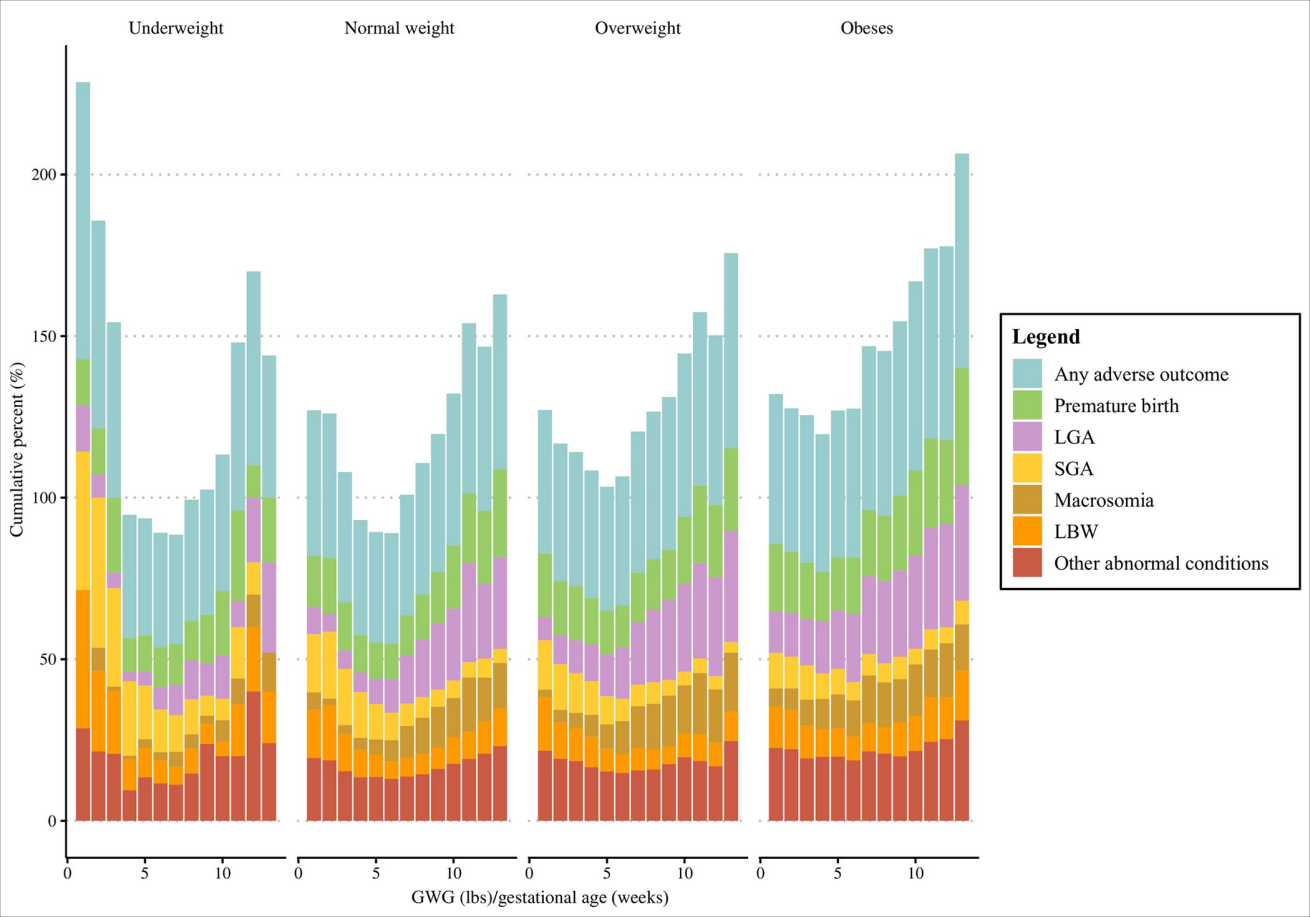
Relationship between average GWG per gestational age and adverse neonatal outcomes in different preconception BMI groups. BMI, body mass index; GWG, gestational weight gain; LGA, large for gestational age; SGA, small for gestational age; LBW, low birth weight.

The overall incidence of any adverse outcome showed a U-shaped trend as GWG increased. The relationship between any adverse outcome and GWG and the relationship between any adverse outcome and average GWG per gestational age had a similar trend. When GWG was within a reasonable range, the incidence of adverse neonatal outcomes may be the lowest (Fig 2). Then analysis based on different preconception BMI groups (underweight, normal weight, overweight, obese groups) showed that in different preconception BMI groups, the associations of GWG and average GWG per gestational age with the incidence of adverse neonatal outcomes were different, indicating that preconception BMI should be considered when classifying GWG (Figs 3 and 4).

### Association between GWG and adverse neonatal outcomes

#### Model 1

Univariate analysis illustrated that compared with sufficient GWG, insufficient GWG and excessive GWG were significantly associated with the risks of any adverse outcome, premature birth, LGA, SGA, macrosomia, and LBW (all *P*<0.05), and insufficient GWG was associated with a significantly increased risk of other abnormal conditions (*P*<0.001) (Table 2).

**Table 2:** Association between GWG and adverse neonatal outcomes.

Outcomes	GWG	Model 1		Model 2		Model 3		Model 4	
		OR (95%CI)	*P*	OR (95%CI)	P	OR (95%CI)	*P*	OR (95%CI)	*P*
Any adverse outcome									
	Sufficient	Ref		Ref		Ref		Ref	
	Insufficient	1.23 (1.19–1.27)	<0.001	1.22 (1.18–1.26)	<0.001	1.22 (1.18–1.27)	<0.001	1.11 (1.07–1.16)	<0.001
	Excessive	1.19 (1.16–1.23)	<0.001	1.20 (1.17–1.23)	<0.001	1.19 (1.16–1.22)	<0.001	1.14 (1.10–1.18)	<0.001
Premature birth									
	Sufficient	Ref		Ref		Ref		Ref	
	Insufficient	1.45 (1.39–1.52)	<0.001	1.43 (1.37–1.50)	<0.001	1.44 (1.37–1.50)	<0.001	1.42 (1.35–1.48)	<0.001
	Excessive	0.95 (0.91–0.98)	0.006	0.96 (0.92–1.00)	0.034	0.95 (0.91–0.99)	0.007	0.86 (0.83–0.90)	<0.001
LGA									
	Sufficient	Ref		Ref		Ref		Ref	
	Insufficient	0.73 (0.69–0.77)	<0.001	0.74 (0.70–0.79)	<0.001	0.74 (0.70–0.79)	<0.001	0.71 (0.66–0.75)	<0.001
	Excessive	1.86 (1.79–1.94)	<0.001	1.83 (1.76–1.90)	<0.001	1.82 (1.75–1.89)	<0.001	1.50 (1.44–1.56)	<0.001
SGA									
	Sufficient	Ref		Ref		Ref		Ref	
	Insufficient	1.54 (1.46–1.62)	<0.001	1.49 (1.42–1.57)	<0.001	1.49 (1.42–1.57)	<0.001	1.45 (1.37–1.53)	<0.001
	Excessive	0.66 (0.63–0.69)	<0.001	0.68 (0.65–0.71)	<0.001	0.68 (0.65–0.72)	<0.001	0.79 (0.75–0.83)	<0.001
Macrosomia									
	Sufficient	Ref		Ref		Ref		Ref	
	Insufficient	0.58 (0.54–0.63)	<0.001	0.60 (0.55–0.65)	<0.001	0.60 (0.55–0.65)	<0.001	0.68 (0.63–0.74)	<0.001
	Excessive	2.13 (2.03–2.24)	<0.001	2.08 (1.97–2.18)	<0.001	2.07 (1.97–2.18)	<0.001	1.60 (1.51–1.69)	<0.001
LBW									
	Sufficient	Ref		Ref		Ref		Ref	
	Insufficient	1.87 (1.77–1.98)	<0.001	1.83 (1.73–1.93)	<0.001	1.83 (1.73–1.93)	<0.001	1.47 (1.37–1.58)	<0.001
	Excessive	0.76 (0.72–0.81)	<0.001	0.79 (0.74–0.83)	<0.001	0.78 (0.74–0.82)	<0.001	0.85 (0.79–0.91)	<0.001
Other abnormal conditions									
	Sufficient	Ref		Ref		Ref		Ref	
	Insufficient	1.32 (1.26–1.37)	<0.001	1.31 (1.26–1.37)	<0.001	1.31 (1.26–1.37)	<0.001	1.32 (1.27–1.39)	<0.001
	Excessive	1.03 (0.99–1.07)	0.106	1.04 (1.00–1.07)	0.070	1.03 (0.99–1.07)	0.166	0.92 (0.88–0.96)	<0.001

Model 1, a univariate model, did not adjust for covariates

Model 2, a multivariate model, adjusted for maternal age at delivery, maternal race, and maternal education level

Model 3, a multivariate model, adjusted for maternal age at delivery, maternal race, maternal education level, paternal age at delivery, paternal race, and paternal education level

Model 4, a multivariate model, adjusted for maternal age at delivery, maternal race, maternal education level, paternal age at delivery, paternal race, paternal education level, parity, smoking before pregnancy, smoking during pregnancy, prenatal care visit, pre-gestational diabetes, gestational diabetes, pre-gestational hypertension, gestational hypertension, hypertension eclampsia, fever, previous premature birth, previous cesarean delivery, and gestational age (gestational age was not adjusted for when premature birth was the outcome).

GWG, gestational weight gain; LGA, large for gestational age; SGA, small for gestational age; LBW, low birth weight; OR, odds ratio; CI, confidence interval; Ref, reference.

#### Model 2

After adjusting for maternal age at delivery, maternal race, and maternal education level, insufficient GWG (OR = 1.22, 95%CI: 1.18–1.26, *P*<0.001) and excessive GWG (OR = 1.20, 95%CI: 1.17–1.23, *P*<0.001) were associated with significantly higher risks of any adverse outcome. Compared with the sufficient GWG group, the insufficient GWG group had significantly increased risks of delivering premature infants (OR = 1.43, 95%CI: 1.37–1.50, *P*<0.001), SGA infants (OR = 1.49, 95%CI: 1.42–1.57, *P*<0.001), LBW infants (OR = 1.83, 95%CI: 1.73–1.93, *P*<0.001), and infants with other abnormal conditions (OR = 1.31, 95%CI: 1.26–1.37, *P*<0.001), and the excessive GWG group had significantly decreased risks of delivering premature infants (OR = 0.96, 95%CI: 0.92–1.00, *P* = 0.034), SGA infants (OR = 0.68, 95%CI: 0.65–0.71, *P*<0.001), and LBW infants (OR = 0.79, 95%CI: 0.74–0.83, *P*<0.001). In contrast to sufficient GWG, insufficient GWG was associated with significantly lower risks of LGA (OR = 0.74, 95%CI: 0.70–0.79, *P*<0.001) and macrosomia (OR = 0.60, 95%CI: 0.55–0.65, *P*<0.001), and excessive GWG was associated with significantly greater risks of LGA (OR = 1.83, 95%CI: 1.76–1.90, *P*<0.001) and macrosomia (OR = 2.08, 95%CI: 1.97–2.18, *P*<0.001) (Table 2).

#### Model 3

After adjusting for maternal age at delivery, maternal race, maternal education level, paternal age at delivery, paternal race, and paternal education level, the risk of any adverse outcome in the insufficient (OR = 1.22, 95%CI: 1.18–1.27, *P*<0.001) and excessive (OR = 1.19, 95%CI: 1.16–1.22, *P*<0.001) GWG groups was significantly higher than that in the sufficient GWG group. Compared with sufficient GWG, insufficient GWG was associated with significantly elevated risks of preterm birth (OR = 1.44, 95%CI: 1.37–1.50, *P*<0.001), SGA (OR = 1.49, 95%CI: 1.42–1.57, *P*<0.001), LBW (OR = 1.83, 95%CI: 1.73–1.93, *P*<0.001), and other abnormal conditions (OR = 1.31, 95%CI: 1.26–1.37, *P*<0.001), while excessive GWG was associated with significantly reduced risks of preterm birth (OR = 0.95, 95%CI: 0.91–0.99, *P* = 0.007), SGA (OR = 0.68, 95%CI: 0.65–0.72, *P*<0.001), and LBW (OR = 0.78, 95%CI: 0.74–0.82, *P*<0.001). Women with insufficient GWG had significantly lower risks of delivering LGA infants (OR = 0.74, 95%CI: 0.70–0.79, *P*<0.001) and macrosomia (OR = 0.60, 95%CI: 0.55–0.65, *P*<0.001), and those with excessive GWG showed significantly greater risks of delivering LGA infants (OR = 1.82, 95%CI: 1.75–1.89, *P*<0.001) and macrosomia (OR = 2.07, 95%CI: 1.97–2.18, *P*<0.001) than women with sufficient GWG (Table 2).

#### Model 4

After adjusting for maternal age at delivery, maternal race, maternal education level, paternal age at delivery, paternal race, paternal education level, parity, smoking before pregnancy, smoking during pregnancy, prenatal care visit, pre-gestational diabetes, gestational diabetes, pre-gestational hypertension, gestational hypertension, hypertension eclampsia, fever, previous premature birth, previous cesarean delivery, and gestational age (gestational age was not adjusted for when premature birth was the outcome), women with insufficient GWG (OR = 1.11, 95%CI: 1.07–1.16, *P*<0.001) and excessive GWG (OR = 1.14, 95%CI: 1.10–1.18, *P*<0.001) had significantly greater risks of any adverse outcome than those with sufficient GWG. In contrast to sufficient GWG, insufficient GWG was associated with significantly elevated risks of premature birth (OR = 1.42, 95%CI: 1.35–1.48, *P*<0.001), SGA (OR = 1.45, 95%CI: 1.37–1.53, *P*<0.001), LBW (OR = 1.47, 95%CI: 1.37–1.58, *P*<0.001), and other abnormal conditions (OR = 1.32, 95%CI: 1.27–1.39, *P*<0.001), and excessive GWG was associated with significantly lower risks of premature birth (OR = 0.86, 95%CI: 0.83–0.90, *P*<0.001), SGA (OR = 0.79, 95%CI: 0.75–0.83, *P*<0.001), LBW (OR = 0.85, 95%CI: 0.79–0.91, *P*<0.001), and other abnormal conditions (OR = 0.92, 95%CI: 0.88–0.96, *P*<0.001). Infants born to women with insufficient GWG had significantly decreased risks of LGA (OR = 0.71, 95%CI: 0.66–0.75, *P*<0.001) and macrosomia (OR = 0.68, 95%CI: 0.63–0.74, *P*<0.001), and infants born to women with excessive GWG had significantly increased risks of LGA (OR = 1.50, 95%CI: 1.44–1.56, *P*<0.001) and macrosomia (OR = 1.60, 95%CI: 1.51–1.69, *P*<0.001) (Table 2).

### Association between GWG and adverse neonatal outcomes in subpopulations

#### Maternal age at delivery subpopulations

**Fig 5:**
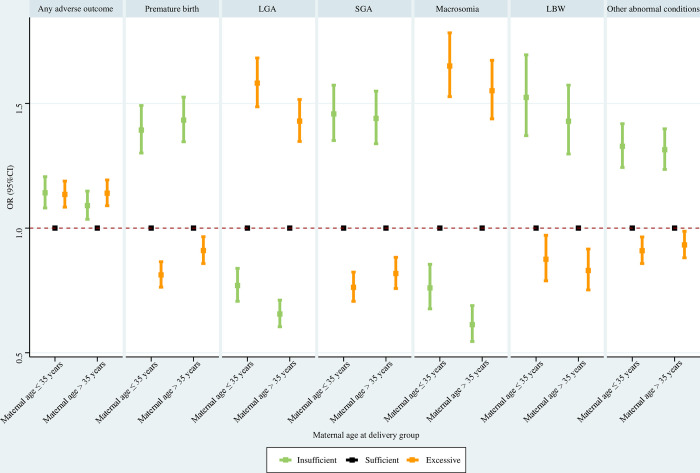
Association between GWG and adverse neonatal outcomes in maternal age at delivery subpopulations. Maternal race, maternal education level, paternal age at delivery, paternal race, paternal education level, parity, smoking before pregnancy, smoking during pregnancy, prenatal care visit, pre-gestational diabetes, gestational diabetes, pre-gestational hypertension, gestational hypertension, hypertension eclampsia, fever, previous premature birth, previous cesarean delivery, and gestational age were adjusted for (gestational age was not adjusted for when premature birth was the outcome). GWG, gestational weight gain; LGA, large for gestational age; SGA, small for gestational age; LBW, low birth weight; OR, odds ratio; CI, confidence interval.

For both women ≤ 35 years and women > 35 years, insufficient GWG and excessive GWG were associated with significantly greater risks of any adverse outcome than sufficient GWG. Among both women ≤ 35 years and women > 35 years, insufficient GWG was associated with significantly increased risks of premature birth, SGA, LBW, and other abnormal conditions, and significantly reduced risks of LGA and macrosomia; excessive GWG was associated with significantly lower risks of premature birth, SGA, LBW, and other abnormal conditions, and significantly higher risks of LGA and macrosomia (Fig 5).

#### Paternal age at delivery subpopulations

**Fig 6:**
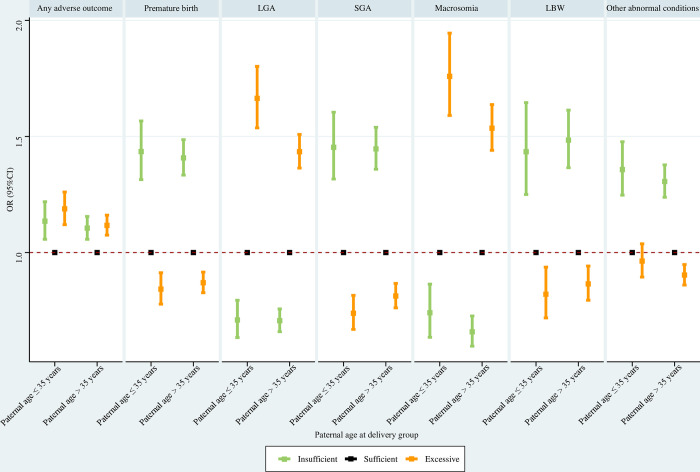
Association between GWG and adverse neonatal outcomes in paternal age at delivery subpopulations. Maternal age at delivery, maternal race, maternal education level, paternal race, paternal education level, parity, smoking before pregnancy, smoking during pregnancy, prenatal care visit, pre-gestational diabetes, gestational diabetes, pre-gestational hypertension, gestational hypertension, hypertension eclampsia, fever, previous premature birth, previous cesarean delivery, and gestational age were adjusted for (gestational age was not adjusted for when premature birth was the outcome). GWG, gestational weight gain; LGA, large for gestational age; SGA, small for gestational age; LBW, low birth weight; OR, odds ratio; CI, confidence interval.

Regarding paternal age ≤ 35 years, insufficient GWG was associated with significantly elevated risks of any adverse outcome, premature birth, SGA, LBW, and other abnormal conditions, and significantly reduced risks of LGA and macrosomia; excessive GWG was associated with significantly higher risks of any adverse outcome, LGA, and macrosomia, and significantly lower risks of premature birth, SGA and LBW. For paternal age > 35 years, insufficient GWG was associated with significantly increased risks of any adverse outcome, premature birth, SGA, LBW, and other abnormal conditions, and significantly decreased risks of LGA and macrosomia; excessive GWG was associated with significantly greater risks of any adverse outcome, LGA, and macrosomia, and significantly lower risks of premature birth, SGA, LBW, and other abnormal conditions (Fig 6).

#### Preconception BMI subpopulations

**Fig 7:**
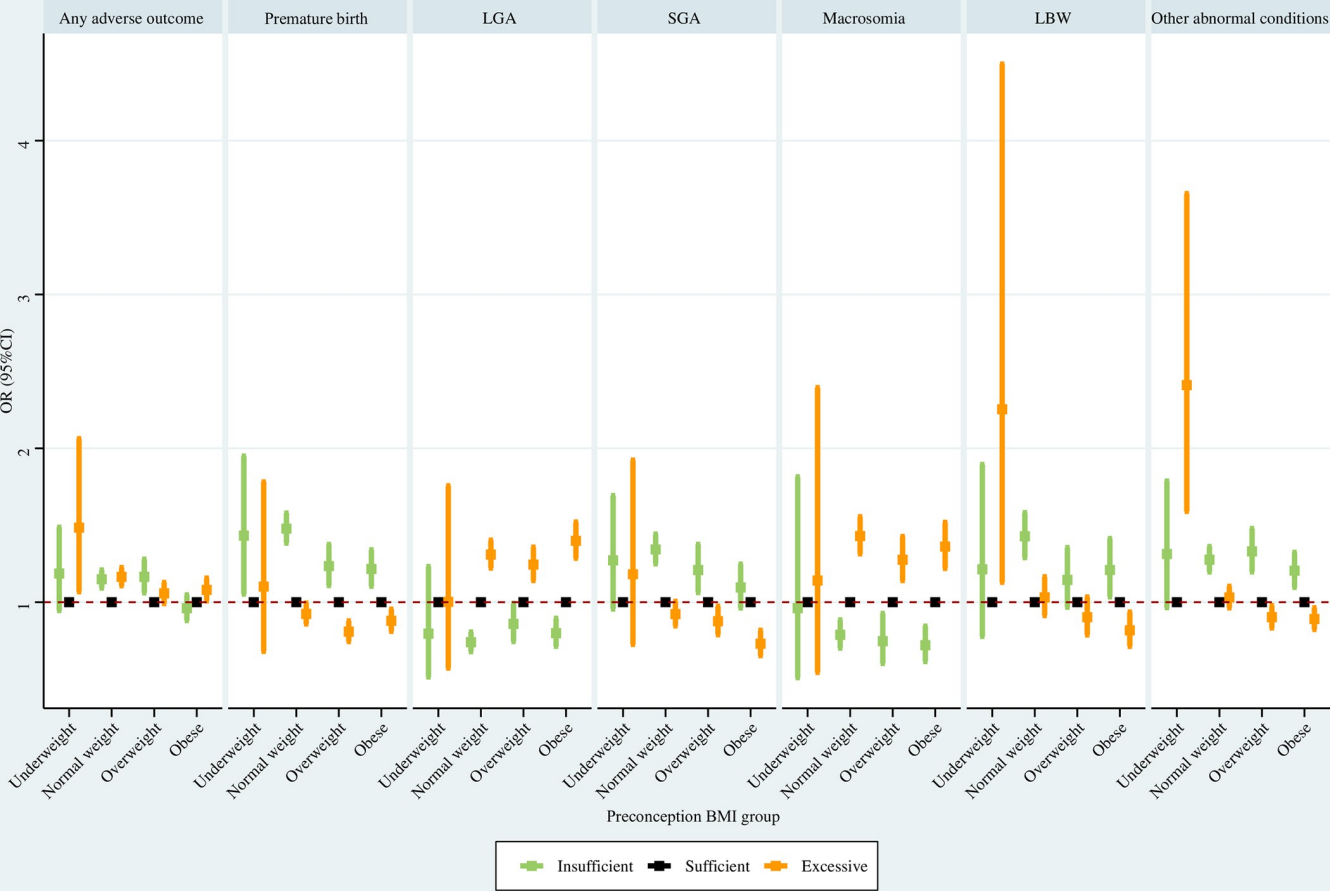
Association between GWG and adverse neonatal outcomes in preconception BMI subpopulations. Maternal age at delivery, maternal race, maternal education level, paternal age at delivery, paternal race, paternal education level, parity, smoking before pregnancy, smoking during pregnancy, prenatal care visit, pre-gestational diabetes, gestational diabetes, pre-gestational hypertension, gestational hypertension, hypertension eclampsia, fever, previous premature birth, previous cesarean delivery, and gestational age were adjusted for (gestational age was not adjusted for when premature birth was the outcome). GWG, gestational weight gain; BMI, body mass index; LGA, large for gestational age; SGA, small for gestational age; LBW, low birth weight; OR, odds ratio; CI, confidence interval.

For underweight women before pregnancy, insufficient GWG was associated with significantly increased risks of premature birth, excessive GWG was associated with significantly higher risks of any adverse outcome, LBW, and other abnormal conditions. For normal weight women before pregnancy, insufficient GWG was associated with greater risks of any adverse outcome, premature birth, SGA, LBW, and other abnormal conditions; excessive GWG was associated with significantly increased any adverse outcome, LGA, and macrosomia. For overweight women before pregnancy, insufficient GWG was associated with significantly increased risks of any adverse outcome, premature birth, SGA, and other abnormal conditions; excessive GWG was associated with a significantly lower risk of premature birth, and significantly higher risks of LGA and macrosomia. For obese women before pregnancy, insufficient GWG was associated with significantly elevated risks of premature birth, LBW, and other abnormal conditions, and significantly decreased risks of LGA and macrosomia; excessive GWG was associated with significantly lower risks of premature birth, SGA, LBW, and other abnormal conditions, and significantly higher risks of LGA and macrosomia (Fig 7).

#### Gestational age subpopulations

**Fig 8:**
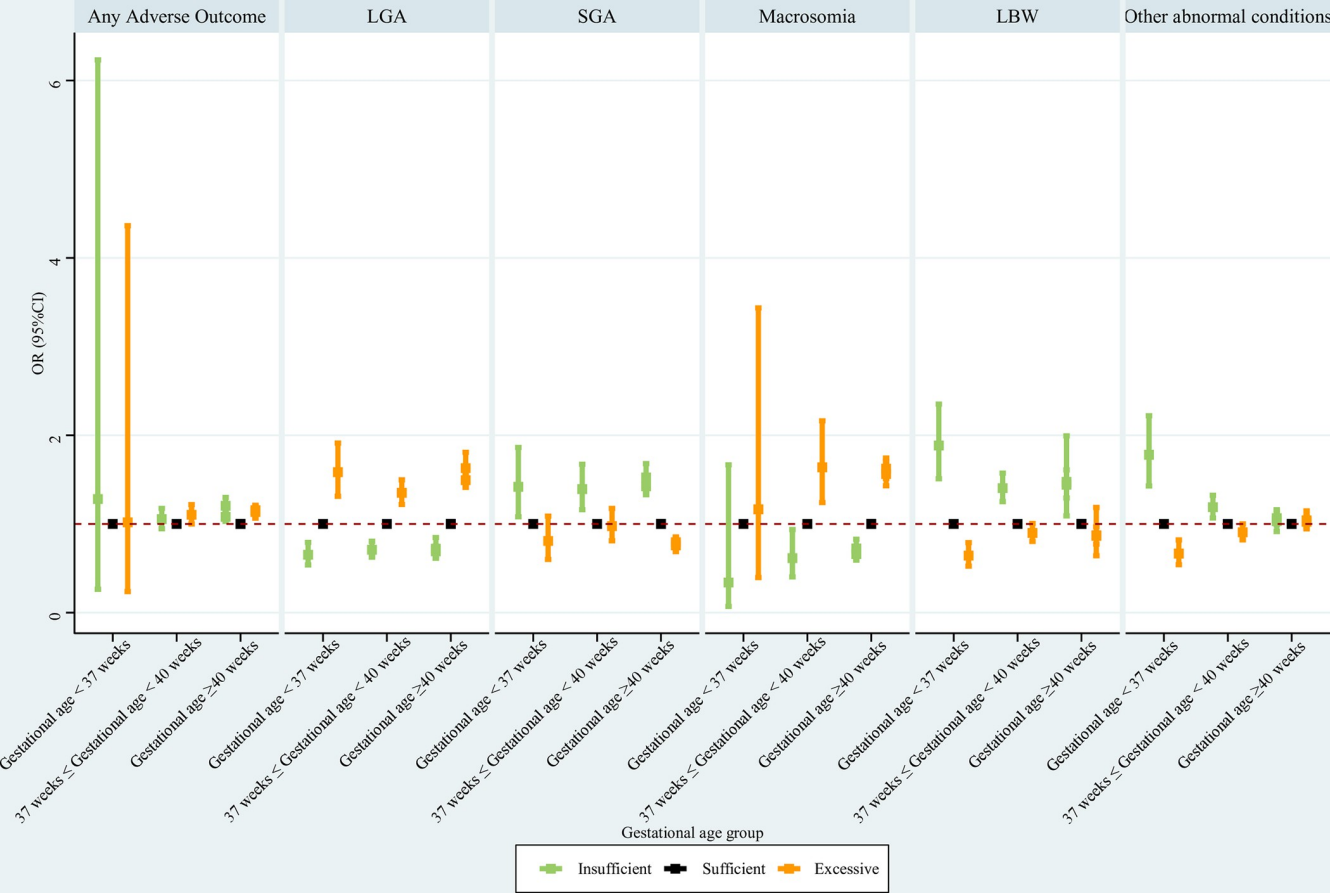
Association between GWG and adverse neonatal outcomes in gestational age subpopulations. Maternal age at delivery, maternal race, maternal education level, paternal age at delivery, paternal race, paternal education level, parity, smoking before pregnancy, smoking during pregnancy, prenatal care visit, pre-gestational diabetes, gestational diabetes, pre-gestational hypertension, gestational hypertension, hypertension eclampsia, fever, previous premature birth, and previous cesarean delivery were adjusted for; premature birth was not assessed in gestational age subpopulations. GWG, gestational weight gain; LGA, large for gestational age; SGA, small for gestational age; LBW, low birth weight; OR, odds ratio; CI, confidence interval.

Concerning women with gestational age <37 weeks, insufficient GWG was associated with a significantly lower risk of LGA, and significantly increased risks of SGA, LBW, and other abnormal conditions; excessive GWG was associated with a significantly elevated risk of LGA, and significantly reduced risks of LBW and other abnormal conditions. For women with gestational age of 37 weeks and above and less than 40 weeks, insufficient GWG was associated with a significantly reduced risk of LGA, and significantly increased risks of SGA, LBW, and other abnormal conditions; excessive GWG was associated with significantly higher risks of LGA and macrosomia. For women with gestational age ≥40 weeks, insufficient GWG was associated with significantly reduced risks of LGA and macrosomia, and significantly higher risks of SGA and LBW; excessive GWG was associated with significantly increased risks of any adverse outcome, LGA, and macrosomia, and a significantly lower risk of SGA (Fig 8).

#### Maternal race subpopulations

**Fig 9:**
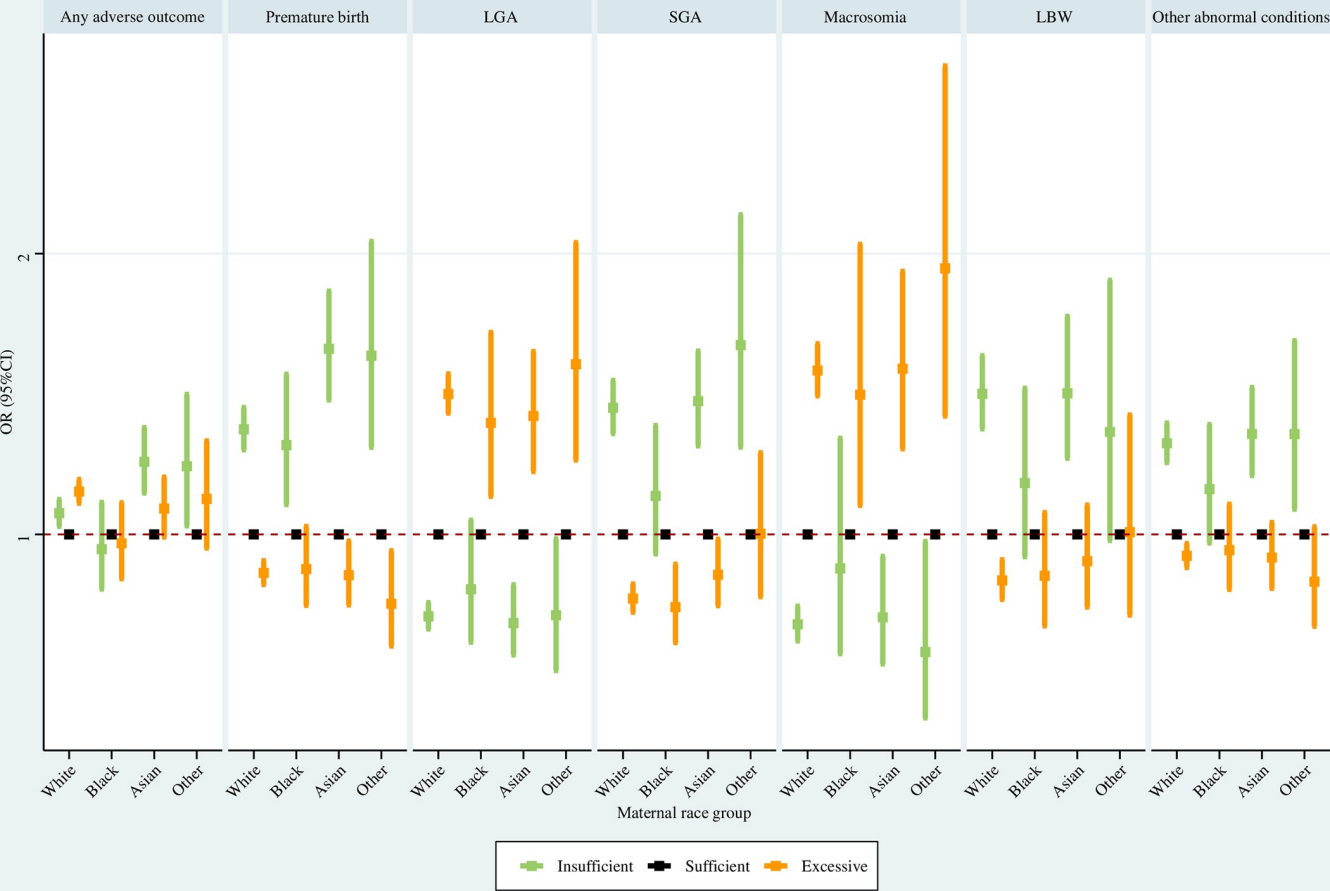
Association between GWG and adverse neonatal outcomes in maternal race subpopulations. Maternal age at delivery, maternal education level, paternal age at delivery, paternal race, paternal education level, parity, smoking before pregnancy, smoking during pregnancy, prenatal care visit, pre-gestational diabetes, gestational diabetes, pre-gestational hypertension, gestational hypertension, hypertension eclampsia, fever, previous premature birth, previous cesarean delivery, and gestational age were adjusted for (gestational age was not adjusted for when premature birth was the outcome). GWG, gestational weight gain; LGA, large for gestational age; SGA, small for gestational age; LBW, low birth weight; OR, odds ratio; CI, confidence interval.

As regards White women, insufficient GWG was associated with significantly greater risks of any adverse outcome, premature birth, SGA, LBW, and other abnormal conditions, and significantly lower risks of LGA and macrosomia; excessive GWG was associated with significantly elevated risks of any adverse outcome, LGA, and macrosomia, and significantly decreased risks of premature birth, SGA, LBW, and other abnormal conditions. For Black women, insufficient GWG was associated with a significantly higher risk of premature birth; excessive GWG was associated with significantly greater risks of LGA and macrosomia, and a significantly lower risk of SGA. For Asian women, insufficient GWG was associated with significantly elevated risks of any adverse outcome, premature birth, SGA, LBW, and other abnormal conditions, and a significantly reduced risk of LGA and macrosomia; excessive GWG was associated with significantly lower risks of premature birth and SGA, and significantly higher risks of LGA and macrosomia (Fig 9).

#### Parity subpopulations

**Fig 10:**
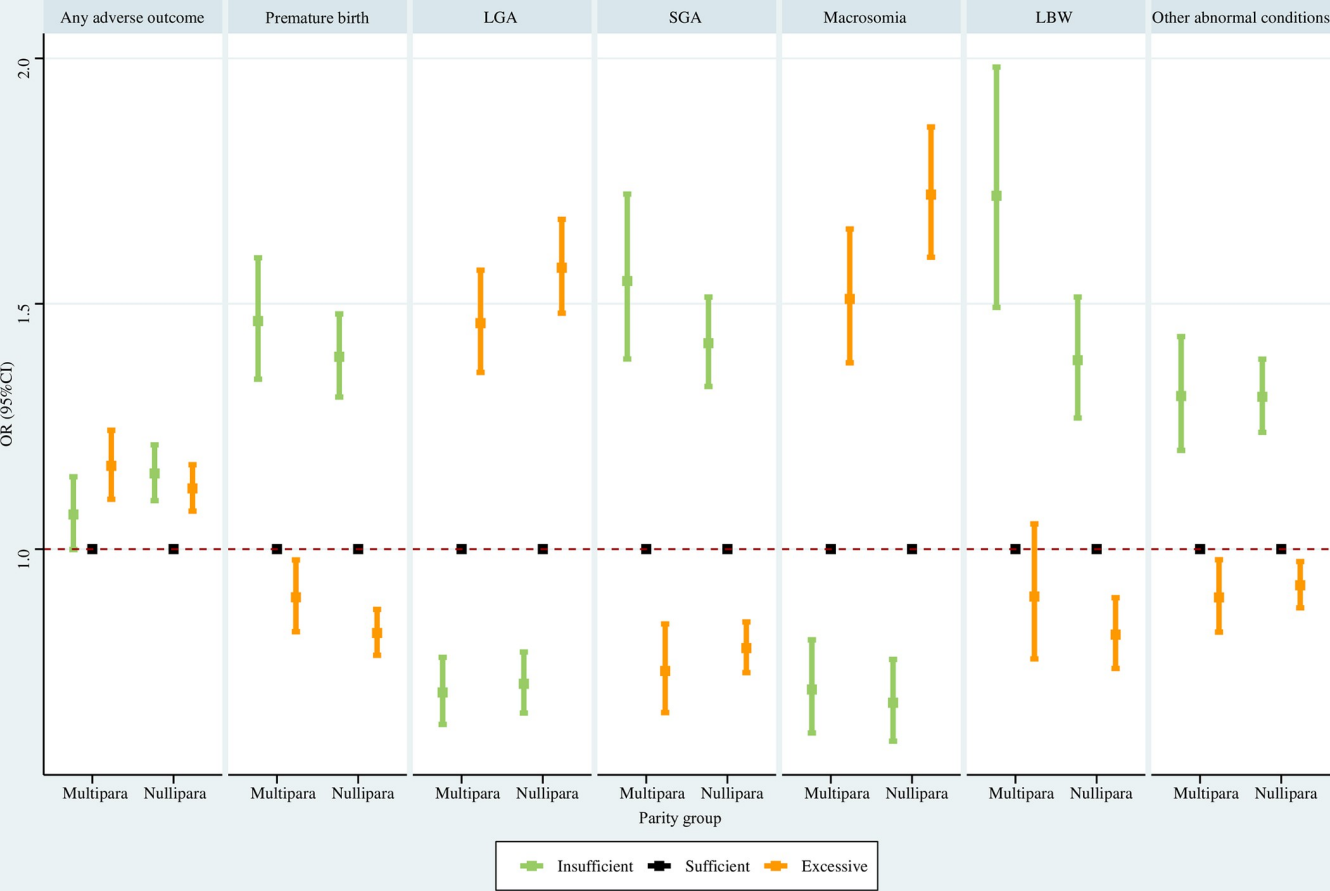
Association between GWG and adverse neonatal outcomes in parity subpopulations. Maternal age at delivery, maternal race, maternal education level, paternal age at delivery, paternal race, paternal education level, smoking before pregnancy, smoking during pregnancy, prenatal care visit, pre-gestational diabetes, gestational diabetes, pre-gestational hypertension, gestational hypertension, hypertension eclampsia, fever, previous premature birth, previous cesarean delivery, and gestational age were adjusted for (gestational age was not adjusted for when premature birth was the outcome). GWG, gestational weight gain; LGA, large for gestational age; SGA, small for gestational age; LBW, low birth weight; OR, odds ratio; CI, confidence interval.

For multiparas, insufficient GWG was associated with significantly higher risks of premature birth, SGA, LBW, and other abnormal conditions, and significantly lower risks of LGA and macrosomia; excessive GWG was associated with significantly greater risks of any adverse outcome, LGA, and macrosomia, and significantly reduced risks of premature birth, SGA and other abnormal conditions. For nulliparas, insufficient GWG was associated with significantly increased risks of any adverse outcome, premature birth, SGA, LBW, and other abnormal conditions, and significantly decreased risks of LGA and macrosomia; excessive GWG was associated with significantly elevated risks of any adverse outcome, premature birth, LGA, and macrosomia, and significantly reduced risks of SGA, LBW, and other abnormal conditions (Fig 10).

#### Gestational diabetes subpopulations

**Fig 11:**
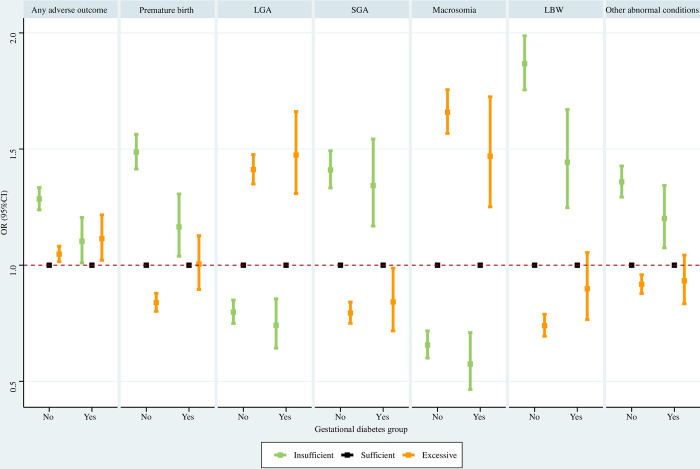
Association between GWG and adverse neonatal outcomes in gestational diabetes subpopulations. Maternal age at delivery, maternal race, maternal education level, paternal age at delivery, paternal race, paternal education level, parity, smoking before pregnancy, smoking during pregnancy, prenatal care visit, pre-gestational diabetes, pre-gestational hypertension, gestational hypertension, hypertension eclampsia, fever, previous premature birth, previous cesarean delivery, and gestational age were adjusted for (gestational age was not adjusted for when premature birth was the outcome). GWG, gestational weight gain; LGA, large for gestational age; SGA, small for gestational age; LBW, low birth weight; OR, odds ratio; CI, confidence interval.

In women without diabetes, insufficient GWG was associated with significantly greater risks of any adverse outcome, premature birth, SGA, LBW, and other abnormal conditions, and significantly lower risks of LGA and macrosomia; excessive GWG was associated with significantly elevated risks of any adverse outcome, premature birth, LGA, and macrosomia, and significantly reduced risks of SGA, LBW and other abnormal conditions. Among women with diabetes, insufficient GWG was associated with significantly higher risks of any adverse outcome, premature birth, SGA, LBW, and other abnormal conditions, and significantly lower risks of LGA and macrosomia; excessive GWG was associated with significantly increased risks of any adverse outcome, LGA, and macrosomia, and significantly decreased risks of SGA (Fig 11).

#### Gestational hypertension subpopulations

**Fig 12:**
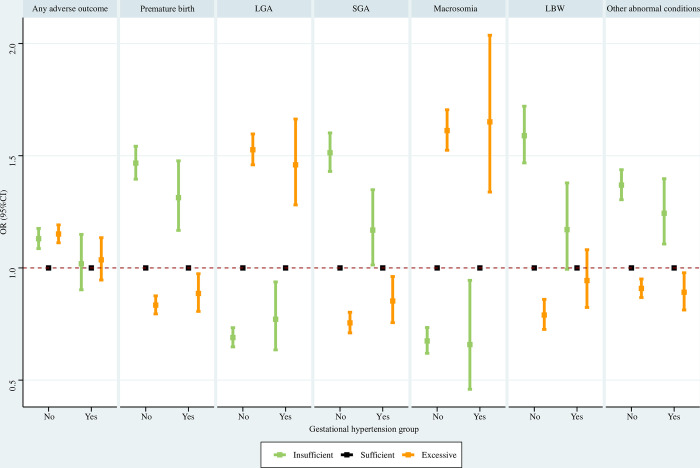
Association between GWG and adverse neonatal outcomes in gestational hypertension subpopulations. Maternal age at delivery, maternal race, maternal education level, paternal age at delivery, paternal race, paternal education level, parity, smoking before pregnancy, smoking during pregnancy, prenatal care visit, pre-gestational diabetes, gestational diabetes, pre-gestational hypertension, hypertension eclampsia, fever, previous premature birth, previous cesarean delivery, and gestational age were adjusted for (gestational age was not adjusted for when premature birth was the outcome). GWG, gestational weight gain; LGA, large for gestational age; SGA, small for gestational age; LBW, low birth weight; OR, odds ratio; CI, confidence interval.

For women without gestational hypertension, insufficient GWG was associated with significantly higher risks of any adverse outcome, premature birth, SGA, LBW, and other abnormal conditions, and significantly lower risks of LGA and macrosomia; excessive GWG was associated with significantly increased risks of any adverse outcome, LGA, and macrosomia, and significantly reduced risks of premature birth, SGA, LBW and other abnormal conditions. For women with gestational hypertension, insufficient GWG was associated with significantly elevated risks of premature birth, SGA and other abnormal conditions, and significantly decreased risks of LGA and macrosomia; excessive GWG was associated with significantly greater risks of LGA and macrosomia, and significantly lower risks of premature birth, SGA and other abnormal conditions (Fig 12).

## Discussion

This study took preconception BMI into account and explored the association between GWG and adverse neonatal outcomes in women who conceived with ART for the first time. With the data on 108201 women from the NVSS, we found that insufficient GWG and excessive GWG were associated with significantly higher risks of any adverse outcome than sufficient GWG, indicating the applicability of the GWG recommended by the IOM in this population. Specifically, compared with sufficient GWG, insufficient GWG was associated with significantly elevated risks of premature birth, SGA, LBW, and other abnormal conditions; excessive GWG was associated with significantly increased risks of LGA and macrosomia. These findings may serve as a reference for weight management of women conceiving via ART to prevent or lower the risk of adverse neonatal outcomes.

According to a systematic review and meta-analysis, insufficient GWG was associated with greater risks of preterm birth and SGA; excessive GWG was associated with higher risks of LGA and macrosomia [14]. Eick et al. [15] reported that women in Puerto Rico with insufficient GWG exhibited an elevated risk of preterm birth. In a systematic review of Han et al. [16], the risks of premature delivery and LBW of singletons born to women with insufficient GWG were higher. Frankenthal et al. [17] found that insufficient GWG was associated with elevated risks of gestational diabetes and SGA, while excessive GWG was associated with a higher risk of pregnancy hypertension in women receiving ART treatments versus those who conceived spontaneously. Another research demonstrated that greater GWG was linked to subsequent preeclampsia among women receiving in vitro fertilization/intracytoplasmic sperm injection (IVF/ICSI) [18]. The current study focused on women who became pregnant via ART, and the association between GWG and adverse neonatal outcomes in this population, the findings of which added to the existing research. Based on the IOM guidelines, for different levels of preconception BMI, the most suitable ranges of GWG (i.e. sufficient GWG) were different. Overall, insufficient GWG and excessive GWG were associated with increased risks of any adverse outcome, which suggested that women could control their weight gain at sufficient GWG levels based on preconception BMI to obtain favorable neonatal outcomes.

With respect to specific neonatal outcomes, insufficient GWG was associated with elevated risks of premature birth, SGA, LBW, and other abnormal conditions; excessive GWG was associated with increased risks of LGA and macrosomia in contrast to sufficient GWG. For potential mechanisms, GWG is regarded as a sign of many physiological processes, including metabolism and body composition, and may indicate nutritional status [19]. Insufficient GWG may reflect macronutrient and micronutrient deficiency, which may cause premature birth and decreased fetal growth [19, 20]. In addition, insufficient GWG may be an intermediate of anemia. Anemia is associated with an elevated risk of premature birth, and about half of women with low weight gain are anemic [15]. Insufficient GWG may stimulate the production of cortisol, which could increase corticotropin-releasing-hormone and prostaglandin, and elevate the susceptibility to uterine contraction [21]. The association between excessive GWG and the risks of LGA and macrosomia may be explained by fetal over-nutrition, because increased nutrient delivery from the placenta to the fetus may lead to an increase in the synthesis of insulin and insulin-like growth factors, both of which are growth-promoting hormones [22].

This study further evaluated the association between GWG and adverse neonatal outcomes among ART conceptions in terms of maternal age at delivery, paternal age at delivery, preconception BMI, gestational age, maternal race, parity, gestational diabetes, and gestational hypertension. For women ≤ 35 years and > 35 years, the associations of insufficient and excessive GWG with all adverse neonatal outcomes were consistent, which suggested that maternal age at delivery did not significantly affect these associations. As regards paternal age at delivery, excessive GWG was associated with a significantly lower risk of other abnormal conditions when paternal age was over 35 years, while no significant was found for paternal age ≤ 35 years, which necessitates more evidence to assess the effect of paternal age on these abnormal conditions. On the other hand, the research by Basso et al. [23] and Ni et al. [24] showed no association of paternal age with the risk of early preterm delivery and average birthweight, which supported the similar relationships between insufficient and excessive GWG and the risks of premature birth, LGA, SGA, macrosomia, and LBW for paternal age ≤ 35 and > 35 years to some extent. For the similar associations between GWG and adverse neonatal outcomes in different maternal and paternal age subpopulations, one explanation is that with the improvement of socioeconomic, cultural and educational environments, couples may develop healthy living habits and have more access to medical assistance and health services for ART pregnancies, which may result in no significant difference among different subgroups. Concerning women with different preconception BMI, the association between GWG and adverse neonatal outcomes varied. As demonstrated by Pongcharoen et al. [25], high pre-pregnancy maternal BMI and excessive GWG were related to an increased risk of infant macrosomia. Normal-weight women with insufficient GWG had a greater risk of delivering SGA neonates [26]. In addition, the association between GWG and adverse neonatal outcomes also differed by gestational age, maternal race, parity, gestational diabetes, and gestational hypertension. These results may facilitate the understanding of subgroup disparities. Medical personnel providing preconception or prenatal care related to adverse neonatal outcomes should consider paternal age at delivery, preconception BMI, gestational age, maternal race, parity, gestational diabetes, and gestational hypertension. Further research is necessary to explore the potential mechanisms for the different associations between GWG and adverse neonatal outcomes. Besides, ART pregnant women in different subpopulations may have their own sufficient weight gain, which requires future studies to determine their appropriate GWG based on the existing data.

The present study utilized a large nationally representative sample from the NVSS, and first evaluated the association between GWG and adverse neonatal outcomes in women conceiving with ART and applicability of the recommended GWG by the IOM in these women. In view of our findings, emphasis should be given on the importance of sufficient GWG for different preconception BMI. For women with preconception BMI < 18.5 kg/m^2^, sufficient GWG is 12.5–18.0 kg; for preconception BMI of 18.5–24.9 kg/m^2^, sufficient GWG is 11.5–16.0 kg; for preconception BMI of 25.0–29.9 kg/m^2^, sufficient GWG is 7.0–11.5 kg; for preconception BMI ≥ 30.0 kg/m^2^, sufficient GWG is 5.0–9.0 kg. Clinicians could provide prenatal counseling for women who conceived via ART to maintain an appropriate weight during pregnancy, and thus manage the risk of adverse neonatal outcomes. Lifestyle interventions, such as having a healthy balanced diet and exercising properly, can help women using ART achieve the recommended GWG. Knowledge about the association between GWG and adverse neonatal outcomes and feasible interventions should also be popularized among this population. Some limitations should be noted when interpreting our findings. First, it is difficult to obtain and adjust for other possible confounders during pregnancy due to the limitation of the NVSS, such as dietary patterns and interventions for pregnancy complications. Second, this study paid attention to singleton pregnancy with 24–42 weeks of gestation, which may not be applicable to multiple pregnancy and newborns outside 24–42 weeks of gestational age. Third, the NVSS were an American database, and our research may have limited generalizability in other populations.

## Conclusion

Insufficient GWG and excessive GWG were associated with increased risks of any adverse outcome than sufficient GWG in women who conceived with ART, indicating the applicability of the GWG recommended by the IOM in this population. These women should maintain an appropriate weight during pregnancy to manage the risk of adverse neonatal outcomes. More studies are needed to confirm our findings.

## Supporting information

S1 Table Sensitivity analysis for data before and after missing value deletion. BMI, body mass index; GWG, gestational weight gain; LGA, large for gestational age; SGA, small for gestational age; LBW, low birth weight; M, Median; Q1, 1st quartile; Q3, 3st quartile.
(DOCX)

S2 Table Univariate analysis for covariate screening. OR, odds ratio; CI, confidence interval Ref, reference.
(DOCX)
