# Supplementary material for: Association between gestational weight gain and adverse neonatal outcomes in women conceiving with assisted reproductive technology: Evidence from the NVSS 2019–2021
Source: PLoS One. 2023 Oct 26;18(10):e0292665. doi: 10.1371/journal.pone.0292665 (PMC10602326; doi:10.1371/journal.pone.0292665)
Supplement: S1 Table — BMI, body mass index; GWG, gestational weight gain; LGA, large for gestational age; SGA, small for gestational age; LBW, low birth weight; M, Median; Q1, 1st quartile; Q3, 3st quartile. (DOCX) [file pone.0292665.s001.docx]

**Supplementary Table 1** Sensitivity analysis for data before and after missing value deletion

| Variables | Total (n=224081) | After deletion (n=108201) | Before deletion (n=115880) | Statistics | *P* |
| --- | --- | --- | --- | --- | --- |
| GWG, n (%) |  |  |  | χ^2^=0.904 | 0.636 |
| Insufficient | 46122 (20.58) | 22282 (20.59) | 23840 (20.57) |  |  |
| Sufficient | 78573 (35.06) | 38034 (35.15) | 40539 (34.98) |  |  |
| Excessive | 99386 (44.35) | 47885 (44.26) | 51501 (44.44) |  |  |
| Preconception BMI, n (%) |  |  |  | χ^2^=9.169 | 0.102 |
| Underweight | 4753 (2.12) | 2315 (2.14) | 2438 (2.10) |  |  |
| Normal | 108141 (48.26) | 52549 (48.57) | 55592 (47.97) |  |  |
| Overweight | 59777 (26.68) | 28719 (26.54) | 31058 (26.80) |  |  |
| Obesity I | 51410 (22.94) | 24618 (22.75) | 26792 (23.12) |  |  |
| GWG, pounds, M (Q_1_, Q_3_) | 29.00 (21.00, 37.00) | 29.00 (21.00, 37.00) | 29.00 (21.00, 37.00) | Z=0.644 | 0.520 |
| Any adverse outcome, n (%) |  |  |  | χ^2^=0.714 | 0.398 |
| No | 132585 (59.17) | 64119 (59.26) | 68466 (59.08) |  |  |
| Yes | 91496 (40.83) | 44082 (40.74) | 47414 (40.92) |  |  |
| Premature birth, n (%) |  |  |  | χ^2^=1.967 | 0.161 |
| No | 190973 (85.22) | 92332 (85.33) | 98641 (85.12) |  |  |
| Yes | 33108 (14.78) | 15869 (14.67) | 17239 (14.88) |  |  |
| LGA, n (%) |  |  |  | χ^2^=0.000 | 0.985 |
| No | 192210 (85.78) | 92810 (85.78) | 99400 (85.78) |  |  |
| Yes | 31871 (14.22) | 15391 (14.22) | 16480 (14.22) |  |  |
| SGA, n (%) |  |  |  | χ^2^=0.074 | 0.786 |
| No | 203567 (90.85) | 98277 (90.83) | 105290 (90.86) |  |  |
| Yes | 20514 (9.15) | 9924 (9.17) | 10590 (9.14) |  |  |
| Macrosomia, n (%) |  |  |  | χ^2^=0.039 | 0.844 |
| No | 205442 (91.68) | 99188 (91.67) | 106254 (91.69) |  |  |
| Yes | 18639 (8.32) | 9013 (8.33) | 9626 (8.31) |  |  |
| LBW, n (%) |  |  |  | χ^2^=1.832 | 0.176 |
| No | 206182 (92.01) | 99645 (92.09) | 106537 (91.94) |  |  |
| Yes | 17899 (7.99) | 8556 (7.91) | 9343 (8.06) |  |  |
| Other abnormal conditions, n (%) |  |  |  | χ^2^=2.136 | 0.144 |
| No | 187531 (83.69) | 90680 (83.81) | 96851 (83.58) |  |  |
| Yes | 36550 (16.31) | 17521 (16.19) | 19029 (16.42) |  |  |

BMI, body mass index; GWG, gestational weight gain; LGA, large for gestational age; SGA, small for gestational age; LBW, low birth weight; M, Median; Q_1_, 1st quartile; Q_3_, 3st quartile.
