# Supplementary material for: Association between gestational weight gain and adverse neonatal outcomes in women conceiving with assisted reproductive technology: Evidence from the NVSS 2019–2021
Source: PLoS One. 2023 Oct 26;18(10):e0292665. doi: 10.1371/journal.pone.0292665 (PMC10602326; doi:10.1371/journal.pone.0292665)
Supplement: S2 Table — OR, odds ratio; CI, confidence interval Ref, reference. (DOCX) [file pone.0292665.s002.docx]

**Supplementary Table 2** Univariate analysis for covariate screening

| Variables | OR (95%CI) | *P* |
| --- | --- | --- |
| Gestational week | 0.61 (0.61-0.62) | <0.001 |
| Maternal age at delivery | 1.01 (1.00-1.01) | <0.001 |
| Maternal race | |  |
| White | Ref |  |
| Black | 1.27 (1.21-1.34) | <0.001 |
| Asian | 0.95 (0.92-0.99) | 0.008 |
| Other | 1.02 (0.96-1.09) | 0.563 |
| Maternal education level | |  |
| High school or above | Ref |  |
| Less than high school | 1.12 (0.98-1.27) | 0.096 |
| Other\unknown | 0.90 (0.84-0.97) | 0.004 |
| Paternal age at delivery | 1.00 (1.00-1.00) | 0.005 |
| Paternal race | |  |
| White | Ref |  |
| Black | 1.24 (1.17-1.30) | <0.001 |
| Asian | 0.95 (0.92-0.99) | 0.013 |
| Other | 0.93 (0.89-0.98) | 0.005 |
| Paternal education level | |  |
| High school or above | Ref |  |
| Less than high school | 1.16 (1.04-1.28) | 0.005 |
| Other\unknown | 0.92 (0.85-0.98) | 0.011 |
| Marital status | |  |
| Married | Ref |  |
| Unmarried | 1.00 (0.94-1.06) | 0.979 |
| Parity |  |  |
| Multipara | Ref |  |
| Nullipara | 1.14 (1.11-1.17) | <0.001 |
| Unknown | 1.08 (1.04-1.13) | <0.001 |
| Smoking before pregnancy | |  |
| No | Ref |  |
| Yes | 1.22 (1.06-1.41) | 0.006 |
| Smoking during pregnancy | |  |
| No | Ref |  |
| Yes | 1.38 (1.11-1.72) | 0.004 |
| Start time of prenatal care | 1.00 (0.98-1.01) | 0.483 |
| Prenatal care visit | 0.97 (0.97-0.97) | <0.001 |
| Pre-gestational diabetes | | |
| No | Ref |  |
| Yes | 1.94 (1.74-2.17) | <0.001 |
| Gestational diabetes | | |
| No | Ref |  |
| Yes | 1.21 (1.17-1.26) | <0.001 |
| Pre-gestational hypertension | |  |
| No | Ref |  |
| Yes | 1.60 (1.50-1.71) | <0.001 |
| Gestational hypertension | |  |
| No | Ref |  |
| Yes | 1.79 (1.73-1.86) | <0.001 |
| Hypertension eclampsia | |  |
| No | Ref |  |
| Yes | 2.73 (2.18-3.40) | <0.001 |
| Fever |  |  |
| No | Ref |  |
| Yes | 1.74 (1.62-1.86) | <0.001 |
| Previous premature birth | | |
| No | Ref |  |
| Yes | 1.61 (1.51-1.72) | <0.001 |
| Previous cesarean delivery | | |
| No | Ref |  |
| Yes | 1.13 (1.09-1.17) | <0.001 |

OR, odds ratio; CI, confidence interval; Ref, reference.
